# The Predictive Validity of the Revised Screening Scale for Pedophilic Interests (SSPI-2)

**DOI:** 10.1177/10790632221149696

**Published:** 2023-02-02

**Authors:** Martina Faitakis, Skye Stephens, Michael C. Seto

**Affiliations:** 1 3653 University of Guelph, Guelph, Canada; 2 3690 Saint Mary’s University, Halifax, Canada; 3 97559 Royal Ottawa Health Care Group, Brockville, Canada

**Keywords:** sexual interests in children, pedohebephilia, SSPI-2, sexual recidivism, screening measure

## Abstract

The Revised Screening Scale for Pedophilic Interests (SSPI-2) is a five-item measure that assesses for pedohebephilia (sexual attraction to prepubescent and pubescent children) based on child victim characteristics. We aimed to replicate findings by Seto, Sandler et al. (2017) by examining the predictive validity of the SSPI-2 in an independent sample of 626 men referred for a sexological assessment because of sexual offending against children. SSPI-2 scores were associated with an increased likelihood of sexual recidivism but were not significantly associated with non-sexually violent or non-violent recidivism. When they were entered together, the SSPI-2 did not contribute additional variance to the Static-99R in the prediction of sexual recidivism. Results are consistent with the findings of Seto, Sandler et al. (2017) and suggest that higher scores on the SSPI-2 may be indicative of an increased risk for sexual recidivism in individuals who have sexually offended against children.

## Introduction

Pedohebephilia is a term used to describe people who have sexual interests in both prepubescent children (pedophilia) and pubescent children (hebephilia) (Blanchard et al., 2009). Although some people who have pedohebephilia perpetrate childhood sexual abuse (CSA), pedohebephilia is not synonymous with sexual offending. In fact, some people who have sexual interests in children do not offend sexually against children and many individuals who have sexually offended against children are not pedohebephilic (Seto, 2005). Nonetheless, sexual interest in children is one of the strongest predictors of sexual recidivism in those who have sexually offended (Hanson & Bussière, 1998; Hanson & Morton-Bourgon, 2004; Mann et al., 2010), so it is an important factor to consider as part of a comprehensive risk assessment. Thus, for people who perpetrate CSA, there is a concerted need to identify whether sexual interest in children is present given its association with reoffending. Several methods can be used to assess sexual interest in children including self-report, phallometric testing (measuring patterns of sexual arousal to sexual stimuli), and sexual offending behavior. The present study is focused on the predictive validity of one of these behavioral measures, the Revised Screening Scale for Pedophilic Interests (SSPI-2), which is a proxy measure of pedohebephilia in men who have perpetrated CSA.

The Screening Scale for Pedophilic Interests (SSPI) is a screening measure developed to assess pedophilia (sexual interest in prepubescent children) based on child victim characteristics (Seto & Lalumière, 2001). The SSPI was created using a sample of 1,113 men with child victims under the age of 15 and is composed of four victim items: any boy victim(s), multiple child victims, child victim(s) under 12 years old, and any extrafamilial child victim(s). The measure was created to be easily scored from file information, including criminal records and clinical interview. In the original development study, the SSPI was associated with sexual arousal to children during phallometric testing. For example, men who scored a five on the SSPI were over five times more likely than those who scored a zero to exhibit greater penile responses to children than to adults (Seto & Lalumière, 2001). Additional research has also suggested that the SSPI has adequate convergent validity with other measures of sexual interest in children (e.g., Banse et al., 2010; Dombert et al., 2017; Helmus et al., 2015; Lehmann et al., 2022; McPhail et al., 2020; Mokros et al., 2010).

In 2017, the Revised Screening Scale for Pedophilic Interests (SSPI-2) was developed based on literature suggesting that offenses related to child pornography^1^ were strongly correlated with pedophilia (Seto, Stephens et al., 2017). With the addition of the new item, the SSPI-2 was better able to classify individuals with phallometrically assessed sexual interest in children than the original SSPI. A subsequent re-analysis of the original data used to create the SSPI-2 found that it was best conceptualized as a measure of pedohebephilia (Stephens et al., 2019).

Given its recent development, there is limited research on the SSPI-2. One study by Renaud (2019) found that as SSPI-2 scores increased, so did the likelihood of clinician diagnosis of pedophilia. For example, 76.8% of those with a SSPI-2 score of four or five were also diagnosed with pedophilia, while only 18.2% of those with a SSPI-2 score of zero had a diagnosis of pedophilia. Additionally, Renaud (2019) reported high inter-rater reliability from scoring the SSPI-2 using medical and clinical records, *r* (20) = .94.

### Predictive Validity of the SSPI and SSPI-2

In the context of risk assessment, predictive validity refers to the ability of an instrument to correctly assess the likelihood of recidivism (see Singh, 2013). Given the importance of sexual interest in children as a risk factor for persistent sexual offending (e.g., Hanson & Morton-Bourgon, 2004), it would be expected that a measure of pedohebephilia, like the SSPI-2, should be associated with a higher likelihood of sexual recidivism. Seto et al. (2004) found the SSPI to be positively correlated with both sexual and violent recidivism, and positively correlated with several validated risk assessment tools (e.g., Static-99, VRAG, SORAG). Another study by Helmus et al. (2015) found that the SSPI was significantly associated with sexual recidivism, similar to other validated risk assessment tools. Although several studies have found the SSPI to have good predictive validity, other research has found that the SSPI was not associated with sexual recidivism (e.g., Canales et al., 2009; Moulden et al., 2009) or had small, non-significant associations with sexual recidivism (McPhail et al., 2020).

Despite research on the SSPI and its predictive validity, there has been limited research on the SSPI-2 and its association with sexual recidivism. Seto, Sandler et al. (2017) examined the concurrent and predictive validity of the SSPI and SSPI-2 in a sample of 2,416 men who had sexually offended against children in New York state. Both the SSPI and SSPI-2 were associated with sexual recidivism (arrest for a sexual offense within 5 years) on their own; the SSPI-2 had slightly better predictive accuracy than the original SSPI, but this apparent difference was not statistically significant. Both measures were negatively associated with any re-arrest, and there was no significant relationship between the SSPI and violent rearrest. This finding indicates that the SSPI and SSPI-2 both seem to be specific indicators of sexual recidivism.

Lastly, research has also examined whether the SSPI and the SSPI-2 add incremental validity to other validated risk assessment measures in the prediction of sexual recidivism. In risk assessment, using more measures does not necessarily make for a better risk prediction (Babchishin et al., 2014; Lehmann et al., 2013; Seto, 2005). On the other hand, Babchishin et al. (2012) found that even highly correlated measures could produce incremental validity in the prediction of sexual recidivism, so it is possible that the SSPI-2 could add incrementally to a significantly correlated measure such as the Static-99R, though it did not in previous studies (Helmus et al., 2015; Seto, Sandler et al., 2017). Therefore, it is important to examine the incremental validity a new measure will have on an existing measure to determine whether it will be useful for clinicians to include in a battery of assessments.

### Present Study

Given that only one previous study to date has examined the predictive validity of the SSPI-2 (e.g., Seto, Sandler et al., 2017), the aim of the present study was to replicate the results for the SSPI-2 in an independent sample of men who had sexually offended against children. It was hypothesized that men with higher scores on the SSPI-2 would be more likely to sexually recidivate than those who scored lower on the SSPI-2 but would not differ in terms of nonsexually violent or nonviolent offenses. We also examined whether the SSPI-2 was a significant predictor when the Static-99R was entered simultaneously; we did not expect this given the results of Seto, Sandler et al. (2017). Given that the SSPI-2 and Static-99R have some overlapping items, we also examined the association between these two measures.

## Method

### Sample

The sample for this study was comprised of men from a large archival database who were assessed at a sexual behavior clinic in Toronto, Canada. This sample includes cases used in the development sample in Seto, Stephens et al. (2017). The initial dataset included 3,343 men who were referred for sexological assessments between 1995 and 2011. For recidivism, criminal records were obtained from the Royal Canadian Mounted Police (RCMP). Criminal records were ordered for 1,117 men in the dataset based on the following inclusion criteria: (a) complete assessment information, (b) valid phallometric testing, (c) assessed after 1995 but before 2006 to ensure adequate follow-up time, (d) at least one victim of any age, and (e) the individual must have been 18 or older at the time of the assessment. From the 1,117 criminal records ordered, only 844 records were obtained, as 273 of the records were not accessible from the national database.

Of the 844 criminal records that were received and coded, 626 were included in the present study based on three further inclusion criteria: (a) the individual must have had at least one child victim under the age of 15, (b) complete information was available in their files to calculate total SSPI-2 and Static-99R scores, and (c) the individual had the opportunity to reoffend in the community after the date of the initial assessment at the sexual behavior clinic. Descriptive statistics for the full sample are presented in Table 1. The hypotheses and planned analyses were pre-registered on Open Science Framework (https://doi.org/10.17605/OSF.IO/FU3VD).

**Table 1.** Descriptive Statistics.

Demographic Variables	*M*(*SD*) or *n*(%)
Average age at assessment (*n* = 626)	41.3 (13.0)
Average age first known offense (*n* = 621)	30.4 (13.0)
Average age at last known offense (*n* = 622)	37.5 (12.6)
Married or common law (*n* = 625)	475 (76.0%)
No known psychiatric history (*n* = 596)	542 (90.9%)
Ethnicity (*n* = 622)	
White	508 (81.7%)
African Canadian	31 (5.0%)
East Indian or Pakistani	14 (2.3%)
Aboriginal Canadian	20 (3.2%)
Asian Canadian	7 (1.1%)
Filipino or Pacific Islander	12 (1.9%)
Ethnicity not listed	30 (4.8%)
Referral (*n* = 609)	
Probation or parole	285 (46.8%)
Correctional institution	191 (31.4%)
Own lawyer	118 (19.4%)
Self- referral	9 (1.5%)
Legal aid	6 (1.0%)

*Note.* For continuous variables, means and standard deviations (in parentheses) are reported and for categorical variables, frequencies and percentages (in parentheses) are reported. Ethnicity labels are consistent with what was recorded at the the time of data collection from the sexual behavior clinic and do not reflect those used by Statistics Canada or the American Psychological Association.

### Measures

#### SSPI-2

The SSPI-2 is a measure of pedohebephilic interests among males who have previously committed a sexual offense against a child (Seto, Stephens et al., 2017; Stephens et al., 2019). It consists of five items scored as present (1) or absent (0): any boy victim(s) under the age of 15, multiple child victims under the age of 15, any child victim(s) under the age of 12, any extrafamilial child victim(s) under the age of 15, and any child pornography offenses. SSPI-2 scores were computed from official file information and self-report. When there was conflicting information about the amount or type of victims between official and self-report sources, the source that had the highest number of victims was used. For example, if an individual admitted to offending towards a boy victim, but official reports only indicated offenses against girl victims, both boy and girl victims would have been coded as present. Scores on the SSPI-2 range from 0 to 5, with higher scores indicating a greater likelihood of pedohebephilia (see introduction for information about its psychometric properties). Total SSPI-2 scores were used as the predictor variable in the Cox regression models.

#### Static-99R

The Static-99R is an actuarial risk assessment used to determine risk of sexual recidivism in adult males who have previously been charged or convicted of at least one sexual offense (Hanson & Thornton, 2000; Helmus et al., 2012). Eight out of the ten items are scored dichotomously: ever lived with an intimate partner, conviction for non-sexual violence at the time of index offense, any convictions for non-sexual violence prior to the index offense, prior sentencing dates, convictions for non-contact sexual offenses, any unrelated victims, any stranger victims, and any male victims. Age at release is scored from 1 to −3, the younger the individual the higher the score. Prior charges and convictions for sexual offenses, is scored from 0 to 3, with a higher score indicating more charges and convictions. Total Static-99R scores can range from −3 (indicating a relatively low risk of reoffending) to 12 (indicating a relatively high risk of reoffending). In terms of psychometric properties, studies have found moderate (AUC = .69; Helmus et al., 2012) to high (AUC = .74; Hanson et al., 2007) predictive validity for the Static-99R and high inter-rater reliability (e.g., ICC = .87; Hanson, 2001; ICC = .90; Barbaree et al., 2001; Quesada et al., 2014). Like the SSPI-2 scores, only total Static-99R scores were used in the analyses.

#### Recidivism

Data for recidivism was collected from the Canadian Police Information Centre (CPIC) of the RCMP, a Canadian federal policing agency. CPIC documents include a federal identifying number for each individual, criminal charges and convictions, and the dispositions for charges in Canada. Recidivism was coded as present when charges or convictions for new offenses that occurred after the initial assessment date had occurred. Recidivism in the original database was coded into four different categories: (a) contact sexual offenses (e.g., sexual assault, aggravated sexual assault), (b) non-contact sexual offenses (e.g., voyeurism, exhibitionism), (c) non-sexual violent offenses (e.g., assault) and (d) non-violent offenses (e.g., theft, drugs). For the present study, contact (10.1%) and non-contact (4.0%) sexual offenses were combined as the main outcome of interest (total sexual recidivism rate was 12.1%) because of the low base rates of non-contact sexual offenses.

Recidivism included new charges or convictions against victims of any age and were dichotomously coded as being present or not present for the purpose of the analyses. The dataset also included the exact end date of the follow-up period for each individual. The follow-up period ended when an individual either died or reoffended, or reached the end of the observation period. If an individual reoffended, the date of the new charge or conviction was used. This is important because Cox regression analyses use “survival time” or the time that it takes from the point of origin until the event of interest occurs or the end of the follow-up period. The event of interest in this case was whether an individual incurred a new charge or conviction for each recidivism outcome.

#### Opportunity to Reoffend

Opportunity to reoffend was operationally defined as the amount of time an individual resided in the community during the follow-up period, while accounting for any time they spent in secure custody. To calculate opportunity to reoffend for each recidivism outcome, a composite score was calculated that involved subtracting the total number of months spent in secure custody since the start of the follow-up period from the total follow-up time for the different types of recidivism. In the Cox regression models, opportunity to reoffend represented the “time to event” or “survival time” that it took for the event of interest to occur.

### Procedure and Data Analysis

The recidivism database used in this study was part of a larger study for the second author’s dissertation. The archival database with victim information was maintained by a research technician who monitored the database for any issues (e.g., to ensure there were no unusual or missing values). The criminal record files were received in September of 2013. A detailed coding manual was created by the second and third authors to code the recidivism data. Both the second author and an experienced research assistant coded the recidivism data, and coding discrepancies between the two were discussed until agreement was reached. The data were also screened at the end of data entry for any significant issues (e.g., out of range values). Inter-rater reliability was also examined between the coders on 10% of the cases and ICC values for each coded variable were found to be above .90, except for violent crime which was .75. Unfortunately, inter-rater reliability was not available for the SSPI-2 because the victim information came from an archival clinical database that was maintained by a research technician at the clinic. Ethics approval was obtained from relevant institutions. The recidivism database has also been used in previous research studies that examined distinct research questions (e.g., Moss et al., 2022; Stephens et al., 2017, 2018)). 

Cox regressions were used to examine the first two hypotheses about the predictive validity of the SSPI-2, and Harrell’s concordance index (Harrell’s C) was calculated as an effect size estimate using R (R Core Team, 2020). For the Cox regression models, the proportional hazards assumption was tested and the data did not violate this assumption. Harrell’s C can be used to estimate the probability that between two randomly chosen individuals, the one with the higher risk score will reoffend before the other and is used when follow-up time is different for each individual, as was the case in this study (Helmus & Babchishin, 2017). Cox regression was also used to examine the third hypothesis, whether the SSPI-2 contributed to the Static-99R in its ability to predict recidivism. We entered both the SSPI-2 and the Static-99R simultaneously into the same block of the regression model instead of testing for true incremental validity. Our decision to do this was consistent with the recommendation of Babchishin et al. (2012) who suggest this approach because highly correlated measures may still produce significant incremental validity in large samples and that incremental validity is typically expected for most risk scales. Given our sample size was quite large (*n* = 626), we chose this method of analysis.

## Results

The rate of any sexual (contact or noncontact) recidivism was 12.1% (*n* = 76). For non-sexually violent recidivism and non-violent recidivism, the rates of recidivism were 11.8% (*n* = 74) and 28.9% (*n* = 181), respectively. The average follow-up time for the sample was 120 months (*SD* = 27.5). For the total sample, the average SSPI-2 score was 2.3 (*SD* = 1.2) and the average Static-99R score was 1.8 (*SD* = 2.4). There was a moderately sized positive correlation between the SSPI-2 and the Static-99R, *r* (624) = .34, *p* < .001, which was expected given the results from Seto, Sandler et al. (2017).

As hypothesized, total SSPI-2 scores were positively associated with an increased likelihood of any sexual recidivism (see Table 2). The hazard ratio for sexual recidivism was 1.27, indicating that a one-point increase on the SSPI-2 was associated with a 27.0% increase in the hazard of any sexual recidivism. Harrell’s C suggested that there was a 57.7% chance that of two randomly selected individuals, the one with the higher SSPI-2 score would sexually recidivate before the other. Consistent with our expectations, the SSPI-2 was not significantly associated with non-sexually violent and non-violent recidivism. Two post hoc Cox regression analyses were conducted by considering contact and non-contact sexual recidivism separately. Results revealed that the SSPI-2 was statistically significantly associated with contact sexual recidivism. SSPI-2 scores were not statistically significant with non-contact sexual recidivism. Although we did not examine the difference in the coefficients between contact and non-contact recidivism, the magnitude of the effect was similar and the confidence intervals overlapped. Hazard ratios and Harrell’s C values are reported in Table 2.

**Table 2.** SSPI-2 Cox Regression Results for Predicting Different Recidivism (*N* = 626).

Predictor	B (SE)	Wald	HR [95% CI]	Harrell’s *C*
Total sexual recidivism	0.24 (0.09)	6.53**	1.27 [1.06, 1.52]	.577
Contact sexual recidivism	0.23 (0.10)	4.92**	1.26 [1.03, 1.53]	.578
Non-contact sexual recidivism	0.31 (0.17)	3.55	1.36 [0.99, 1.89]	.591
Nonsexually violent recidivism	−0.04 (0.09)	0.18	0.96 [0.80, 1.15]	.526
Non-violent recidivism	0.05 (0.06)	0.78	1.06 [0.94, 1.19]	.515

*Note.* Each row represents a separate cox regression model with the SSPI-2 included as the predictor variable.

**p < .01.

SE = standard error; HR = hazard ratio; CI = confidence interval.

Lastly, we found that the SSPI-2 did *not* contribute to the prediction of sexual recidivism when the Static-99R was accounted for in the model. Results of the Cox regression indicated that the overall model was significant, χ^2^(2, 626) = 18.80, *p* < .001, Harrell’s C = .642. While the Static-99-R contributed significantly to the model (*B* = .16, *SE* = .05, *p* < .001), the SSPI-2 did *not* (*B* = .13, *SE* = .10, *p* = .206). For the Static-99R, the hazard ratio was 1.18, 95% CI [1.08, 1.29] indicating that a one-point increase on the Static-99R was associated with a 18.0% increase in the hazard of sexual recidivism. For the SSPI-2, the hazard ratio was 1.14, 95% [0.93, 1.38] indicating that a one-point increase on the SSPI-2 was associated with a 14.0% increase in the hazard of sexual recidivism. Harrell’s C for the overall model was 64.2%.

## Discussion

The goal of this study was to examine whether the SSPI-2 was associated with an increased risk of sexual recidivism, given that only one previous study by Seto, Sandler et al. (2017) has examined this research question. We replicated the positive association between the SSPI-2 and sexual recidivism. We also showed that the effect is specific to sexual recidivism because SSPI-2 scores were not associated with nonsexually violent or non-violent recidivism. Conceptually these results are intuitive, in that the SSPI-2 was not developed to assess for antisociality or general criminality, which is a stronger risk factor for non-sexual offending in men who have sexually offended against children (Brouillette-Alarie et al., 2016). Given that research on the predictive validity of the SSPI (without the child pornography item) has produced mixed results, our findings show promise that the addition of the fifth item (child pornography) may help to improve the SSPI-2’s capacity for predicting sexual recidivism. Since this is only based on findings from this study and Seto, Sandler et al. (2017), more research is needed. Lastly, the SSPI-2 did not contribute beyond the predictive accuracy offered by the Static-99R, which is consistent with past findings as well (e.g., Helmus et al., 2015; Seto, Sandler et al., 2017).

These findings highlight the role that sexual interest in children plays as a risk factor for persistent sexual offending**.** Consistent with Stephens et al. (2017), the effect size for sexual contact recidivism was small. It is also worth noting that the effect size for non-contact sexual recidivism was also small in magnitude. One possibility for the lack of significance for non-contact sexual recidivism may be attributed to the low base rate of non-contact sexual recidivism. The ability of the SSPI-2 to predict non-contact sexual recidivism, especially child pornography offending, should be examined with larger samples in future research. It is also worth reminding readers that the SSPI-2 can only be used with individuals who have perpetrated contact sexual offenses against a victim under the age of 15 or noncontact offenses that involve an interaction with a child victim (e.g., indecent exposure to a child). The SSPI-2 cannot be scored in people who perpetrate child pornography offenses only or who have offended against older victims. Overall, the results are consistent with findings that suggest a small effect for the association between sexual interest in children and sexual recidivism (Mann et al., 2010; McPhail et al., 2019).

As expected, though the SSPI-2 was found to be correlated with the Static-99R, the SSPI-2 did *not* contribute to the Static-99R in its ability to predict sexual recidivism. These findings are consistent with past research that has found the measures to be correlated but did not find that the SSPI or SSPI-2 added incrementally to the Static-99R (Helmus et al., 2015; Seto, Sandler et al., 2017). One reason that the SSPI-2 may not contribute additional variance beyond the Static-99R is due to the overlap in constructs that exists between the two measures. Helmus et al. (2015) noted that two items from the Static-99R are indicators of atypical sexual interests: having a male victim and any unrelated victims. Although it should be noted that these items apply to victims of any age, not only child victims, which differs from the SSPI and SSPI-2. At the same time, the Static-99R has other items that capture sexual criminality and general criminality (Allen & Pflugradt, 2014; Brouillette-Alarie & Proulx, 2013). Given the Static-99R is a broader measure of risk factors for sexual recidivism, it is not surprising that the SSPI-2 does not remain significant when the Static-99R is accounted for.

Despite the consistent finding that the SSPI-2 does not enhance risk prediction beyond established tools, the SSPI-2 might be able to be used as a measure of sexual interest in children on existing risk scales. The original SSPI is associated with the deviant sexual interest items on the STABLE-2007 (Hanson et al., 2007; Helmus et al., 2015) as well as the sexual deviance items on the Violence Risk Scale-Sexual Offense Version (VRS-SO; McPhail et al., 2020). Furthermore, the SSPI can be used on the Sex Offender Risk Appraisal Guide (SORAG) as a substitute for the phallometric item to indicate an individual’s sexual preference (Harris et al., 2015). Therefore, although not a risk assessment measure, Helmus et al. (2015) suggested that the SSPI could be used as a substitute for assessing the sexual deviance items on different risk assessment measures, such as the STABLE-2007. Our findings support this conclusion as well and future research might examine whether the SSPI-2 is a stronger substitute than the SSPI or other measures of sexual interest in children for the pedohebephilia items on established risk assessment tools.

### Limitations

There are several limitations in this study worth mentioning. First, it is acknowledged that the victim characteristics coded from the dataset do not accurately capture all instances of sexual offending against children, as sexual abuse is significantly underreported (Conroy & Cotter, 2017). Therefore, it is assumed this data reflects underreporting of sexual offenses against children, which could have impacted the total number of victims captured in the SSPI-2 scores.

Another limitation is that only CPIC documents were used to identify recidivism. Although CPIC records are meant to include all charges and convictions received anywhere in Canada, there can be discrepancies between CPIC and provincial documents. If available at the initial time of coding, it would have been best to use provincial documents and CPIC documents to code recidivism. Because CPIC documents were the only source used as a measure of recidivism, there could have been offenses not officially detected by police, so the recidivism rates are likely underestimates. It also would have been useful at the time of coding to capture sexual recidivism in a more granular matter, particularly for non-contact sexual recidivism. In the present study, non-contact sexual recidivism included a range of different offenses (e.g., voyeurism, child pornography). Given that child pornography offenses are correlated with pedophilia (Seto, Stephens et al., 2017), it would have been interesting to examine if the SSPI-2 was associated with this type of recidivism more specifically.

Lastly, a more diverse sample would have allowed for better generalizability of the results. Given that most of the sample in this study were White (81.7%), a sample with more ethnic diversity would be beneficial. Results from this study are also only generalizable to men who sexually offend against children; we are not able to draw any conclusions about women or juveniles who offend against children. To date, no study has examined whether the SSPI or the SSPI-2 could be used to assess predictive validity for sexual recidivism among juveniles, which is an area for future research.

### Future Research Directions

Future research should examine the predictive validity of the SSPI-2 with a more diverse sample that includes individuals from outside of North America. Furthermore, based on the nature of the SSPI-2 items (i.e., all based on child victims), it is likely that the results of this study would be most relevant to continued offending against child victims; however, we were unable to tease apart whether new offenses were committed against adults or children. Therefore, it would have been useful to have more accurate descriptive victim information for the recidivism charges and the kinds of charges as well (e.g., age of victim, type of non-contact and contact sexual offenses). For example, having access to police documents would more fulsomely capture arrests and provide details about the offense to allow for more nuanced recidivism coding. Future research on the predictive validity of the SSPI and SSPI-2 would benefit from having access to complete police information and could also examine the predictive validity of the SSPI-2 for offenses specifically perpetrated against children.

## Note

1 The term ‘child pornography’ is used to refer to child sexual exploitation material. The authors understand that ‘child pornography’ does not acknowledge that the material is sexual exploitation. The term ‘child pornography’ is used since this is the way it is referred to in the SSPI and SSPI-2 and it captures the legal definition in the *Canadian Criminal Code* which includes the making, distribution, possession, or access of explicit sexual activity with a person under the age of eighteen.
